# Feasibility of Neurorehabilitation Using a Hybrid Assistive Limb for Patients Who Underwent Spine Surgery

**DOI:** 10.1155/2018/7435746

**Published:** 2018-07-10

**Authors:** Aya Yatsugi, Takashi Morishita, Hiroyuki Fukuda, Naoya Kotani, Kenji Yagi, Hiroshi Abe, Etsuji Shiota, Tooru Inoue

**Affiliations:** ^1^Department of Neurological Surgery, Fukuoka University Faculty of Medicine, Fukuoka, Japan; ^2^Department of Rehabilitation Medicine, Fukuoka University Hospital, Fukuoka, Japan

## Abstract

Recent studies of robotic rehabilitation have demonstrated its efficacy for neurological disorders. However, few studies have used the Hybrid Assistive Limb (HAL) during the early postoperative stage of spine disorders. We aimed to evaluate the safety and efficacy of HAL treatment during the early postoperative period for spine disorder patients. We retrospectively identified patients who underwent spine surgery and who could complete HAL treatment. We evaluated the 10-m walking test (10MWT), the modified Gait Abnormality Rating Scale (GARS-M), Barthel Index (BI), and the walking index for spinal cord injury II (WISCI II) score results before and after robotic rehabilitation. Clinical outcomes were compared after treatment. We included nine patients with various spine problems. After HAL treatment, the speed during the 10MWT significantly improved from 64.1 ± 16.0 to 74.8 ± 10.8 m/min, and the walking cadence decreased from 102.7 ± 17.6 to 92.7 ± 10.9 steps/min. The BI score also improved from 83.3 ± 16.0 to 95.6 ± 5.8, and the WISCI II score improved from 19.7 ± 0.5 to 20.0 ± 0.0. Furthermore, the total GARS-M score improved from 6.0 ± 5.7 to 2.3 ± 3.3. The maximum angles of the trunk swing were improved from 2.2 ± 1.9 to 1.2 ± 0.9 degrees. Neurorehabilitation therapy using HAL for spinal surgery patients was considered feasible following spine surgery.

## 1. Introduction

Robotic technologies have been increasingly gaining attention in the field of neurorehabilitation. The Hybrid Assistive Limb (HAL) (Cyberdyne Inc., Ibaraki, Japan) is a unique exoskeleton robot for neurorehabilitation that was developed by Sankai and colleagues based on the interactive biofeedback (iBF) theory [1, 2]. HAL has a hybrid system that allows both voluntary and autonomous modes of action to support training, and it supports voluntary muscle movement by detecting bioelectrical signals (BES). For walking training, movements of the joints are accurately adjusted by the pressure sensor in the foot bottom and joint angle sensors of the frame. Based on the input information, four actuators of the hip and knee joint are controlled independently [3]. Movements of the affected limbs supported by the HAL system generate sensory feedback to the brain (i.e., iBF) and accelerate motor learning in the process of functional recovery.

Recent studies have shown the safety and efficacy of rehabilitation using HAL robotics for various disorders, including stroke [3–7], spinal cord injury (SCI) [8–14], and quadriceps arthrogenic muscle inhibition [15]. Another recent study demonstrated neuroplasticity induced by HAL treatment [16, 17]. However, five case reports have focused on the efficacy of HAL therapy for postoperative thoracic ossification of the posterior longitudinal ligament (OPLL) [18–22]. These reports indicated that HAL was used as a last resort for gait recovery during the almost chronic phase of the postoperative state [21, 22], and the authors recommended starting HAL-assisted training during the early stage following surgery. Neurorehabilitation during the postoperative state is essential for returning to social activities and preventing disuse syndrome. Based on the findings indicated by these five case reports [18–22], in addition to the reports demonstrating the efficacy of HAL training for SCI cases [8–14], we hypothesized that using HAL may facilitate early recovery after spine surgery. Therefore, we aimed to evaluate the safety and efficacy of HAL-assisted rehabilitation for spine disorder patients during the early postoperative period.

## 2. Materials and Methods

### 2.1. Patient Selection and Study Design

We performed a retrospective chart review of patients with spine disorders treated at our neurosurgical department from October 2011 to February 2016. To evaluate the effects of HAL treatment for improvements in gait, we included patients who could complete HAL treatment at least three times. Additionally, because voluntary muscle contractions are required to gain assistance from the HAL system, we excluded patients with complete or nearly complete paralysis. The protocol of the present study was approved by our institutional review board (IRB), and HAL treatment was performed after receiving written informed consent from each patient.

**Figure 1:**
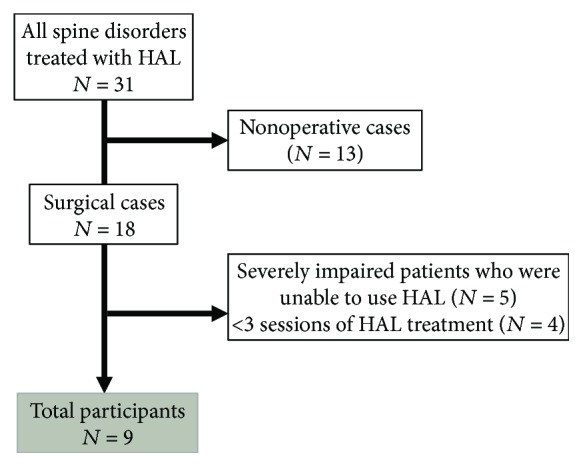
Patient selection flowchart.

HAL treatment was performed for 31 patients with spine disorders; however, 22 patients did not meet the inclusion criteria of the current study (Figure 1). Among those 22 patients, 13 did not undergo surgery.

We investigated the remaining nine patients (six male patients and three female patients) with the following characteristics: severe impairment resulting in the inability to use HAL (*n* = 5) and less than three sessions of HAL treatment (*n* = 4). The mean age of the cohort was 53.6 years (SD, ±16.1). Diagnoses were dural arteriovenous fistula (AVF) (*n* = 2), cervical ossification of the posterior longitudinal ligament (OPLL) (*n* = 1), spinal lipoma (*n* = 1), arachnoid cyst (*n* = 1), spinal ependymoma (*n* = 3), and cervical spondylosis (*n* = 1). Spine lesion levels are summarized in Table 1.

### 2.2. Rehabilitation Program

We performed conventional physical therapy in addition to HAL treatment. Conventional physical therapy started within 2 days after surgery. Depending on the patient's condition, the programs included manual leg stretching, muscular workouts, and basic movement training such as standing, walking, and going up and down stairs. When patients felt fatigue during the HAL treatment, they were allowed to rest. Each session lasted approximately 50 minutes, including time necessary for robotic attachment, and was performed two or three times per week.

HAL treatment started when the patients were able to sit stably. On average, intervention with therapists and HAL treatment began 14.2 ± 8.1 days (range 7–29 days) after surgery. The mean number of HAL treatment sessions was 5.0 ± 2.6 (range 3–12). Rehabilitation periods comprised 13.6 ± 9.1 days (range 4–35 days) during hospitalization at our institution (Table 1).

A bilateral leg version of HAL was used for patients involved in this study (HAL for Living Support–Lower Limb; Cyberdyne Inc.). Training started with the Cybernic Voluntary Control mode, which measures BES from the extensor and flexor muscles of the hip and knee. HAL treatment was performed by one or two physiotherapists and a medical doctor who were trained to use the HAL system. During gait training, the physiotherapist checked the BES and adjusted the HAL assist level.

**Figure 2:**
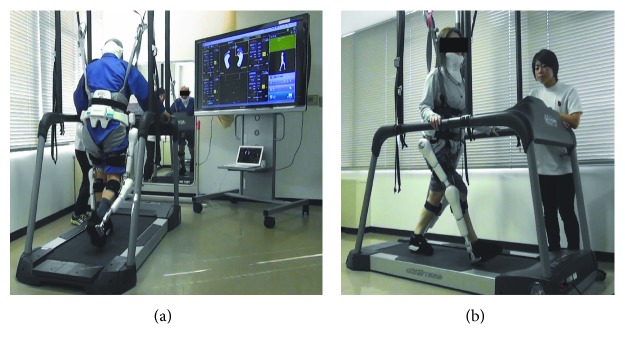
(a, b) Hybrid Assistive Limb (HAL) treatment. Gait training on a treadmill in front of a large monitor.

During HAL treatment, several sets of a knee extension movement were performed (10 times with the left leg and 10 times with the right leg). The standing movement was performed 10 times. Balance training was performed for several seconds with open eyes or closed eyes so that the center of gravity would be in the middle. Finally, walking training was performed on a flat ground or a treadmill. During balance training and gait training, we used a monitor displayed in front of the patient to provide visual feedback regarding the center of gravity, posture, and balance (Figure 2).

We focused on walking training. We used a walk aid called All-In-One Walking Trainer (Ropox A/S, Naestved, Denmark) to secure the safety of patients when walking on a flat floor. It is able to support body weight and enables safe HAL treatment with the use of a harness. We did not use body weight support. After the patient became accustomed to walking on the ground, we began treadmill walking. Patients performed several sets of 5 minutes of walking at a speed that was comfortable with HAL. If patients wanted to continue and were not fatigued, then we increased the speed or increased the walking time. When there was deflection of the center of gravity (it does not take weight to walk on tiptoes), we instructed the patient to move the weight from the heel to the tiptoes.

### 2.3. Outcome Measures

All patients were video-recorded during rehabilitation. The speed and steps during the 10-m walking test (10MWT) as an evaluation of motor function at the time of treatment immediately before wearing HAL and during the last training session after excluding HAL were used to evaluate HAL treatment times. We used the modified Gait Abnormality Rating Scale (GARS-M) [23], the Barthel Index (BI), and the walking index for spinal cord injury II (WISCI II) to evaluate walking appearance, activities of daily living (ADL), and the patients' ambulatory walking capacity on the basis of the need for physical assistance and assistive devices, respectively [24]. The GARS-M includes variables that provide a description of gait associated with an increased risk of falling. The GARS-M considered the following seven items: (1) variability, (2) guardedness, (3) staggering, (4) foot contact, (5) hip range of motion (ROM), (6) shoulder extension, and (7) arm–heel strike synchrony. Each item of the GARS-M is rated from 0 to 3, with a maximum of 21 points; a score of 21 points indicates the worst state. We measured the maximum angle of the trunk swing during the 10MWT before and after treatment using the Total Motion Coordinate System version 3.28 (Toso System Inc., Tokyo, Japan) motion analysis device.

### 2.4. Statistical Analysis

We performed a paired *t*-test to compare the clinical outcomes and baseline. We used SPSS version 21.0 (IBM Corp., Armonk, NY, USA) for the analyses. The mean ± SD values are described.

## 3. Results

**Figure 3:**
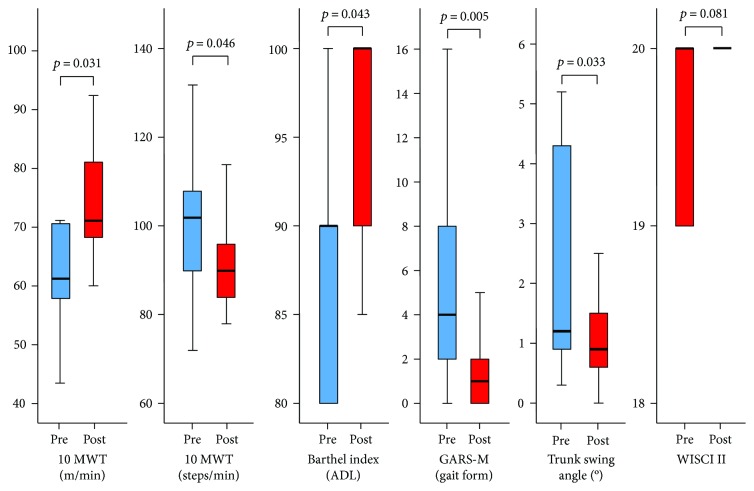
Graph showing functional outcomes evaluated by the 10-m walking test (10MWT): speed (m/min), cadence (steps/min), the Barthel Index, modified Gait Abnormality Rating Scale (GARS-M), trunk swing angle before and after Hybrid Assistive Limb (HAL) treatment, and the walking index for spinal cord injury II (WISCI II) score. Whiskers represent the standard deviation. Pre: before treatment; Post: after treatment.

After HAL treatment, the speed during the 10MWT significantly improved from 64.1 ± 16.0 to 74.8 ± 10.8 m/min (*P* = 0.031), and the cadence decreased from 102.7 ± 17.6 to 92.7 ± 10.9 steps/min (*P* = 0.046). The BI score also improved from 83.3 ± 16.0 to 95.6 ± 5.8 (*P* = 0.043). Furthermore, the total GARS-M score improved from 6.0 ± 5.7 to 2.3 ± 3.3 (*P* = 0.005). The maximum angles of the trunk swing were improved from 2.2 ± 1.9 to 1.2 ± 0.9 degrees (*P* = 0.033). The WISCI II score also improved from 19.7 ± 0.5 to 20.0 ± 0.0 (*P* = 0.081). These scores are summarized in Figure 3. There were no adverse events due to HAL treatment such as pain and/or falling.

It is noteworthy that almost all subjects had improved gait posture. After reviewing each item before and after HAL treatment, it became clear that the subscores of guardedness (item 2), staggering (item 3), and shoulder extension (item 6) showed the most dramatic improvements. Momentum and the ability to move the legs forward were improved. Collapse of balance toward the side was decreased. The movement range of the shoulder toward the backside was expanded.

### 3.1. Representative Case (Case 2)

**Figure 4:**
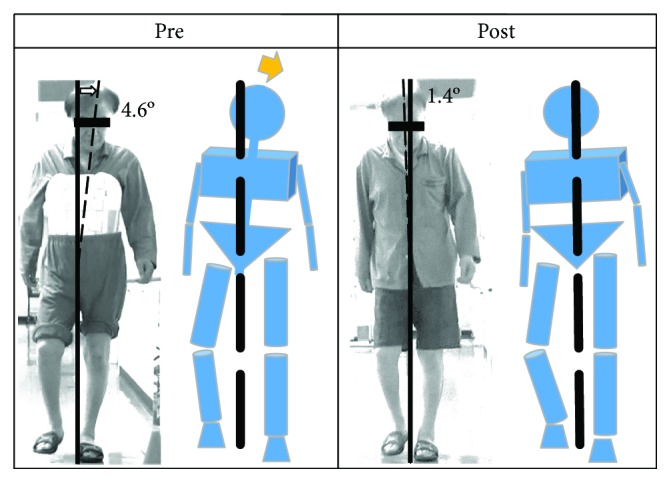
Representative case showing improvements in walking appearance. Prior to Hybrid Assistive Limb (HAL) treatment, the trunk leaned toward the left when standing with the left leg. Furthermore, the trunk was leaned 4.6° to the left. After 12 sessions of HAL treatment, the upper body was stabilized. Pre: before training; Post: after training.

A 65-year-old man was diagnosed with dural AVF at the level of Th6-7 and underwent laminectomy for ligation of the draining vein. Preoperatively, he had urinary continence and was wheelchair-bound. A few days after surgery, conventional physical therapy was started and his walking ability gradually improved so that he could walk with an aid on postoperative day 9. However, his gait posture had involved sweeping out his lower limbs at the cost of laterally bending the trunk to the opposite side (Figure 4). He also had difficulty in kicking the ground with the toes.

We started using HAL on postoperative day 13. At this point, his WISCI II score was 19. At first, he performed knee extension movements and standing training. Next, he started balance training and gait training with a walking device (All-In-One Walking Trainer; Ropox A/S). Later, walking training on a treadmill was initiated.

Before HAL treatment, his trunk was bending forward and he required walking support. After 12 sessions of HAL treatment, the trunk lifted while walking and posture improved. He could constantly set the position of his foot and the step width. He became able to kick the ground on tiptoes and swing out his lower limbs without side bending of the trunk. The angle of his trunk swing during 10MWT decreased from 4.6 to 1.4 degrees (Figure 4). His 10MWT speed improved from 43.4 to 65.7 m/min, and the walking cadence decreased from 132 to 96 steps/min. The total GARS-M score improved from 16 to 10. Similarly, the BI and WISCI II score improved from 55 to 100 and from 19 to 20, respectively.

## 4. Discussion

The HAL was invented based on the iBF theory [1, 2] that movements of the affected limbs supported by the HAL system generate sensory feedback to the brain (i.e., iBF) and accelerate motor learning in the process of functional recovery. HAL therapy may address spasticity due to central nervous system (CNS) lesions. A CNS lesion above the level of the central pattern generator (CPG) results in a loss of supraspinal drive and spasticity. The consequences are hyperexcitability of short-latency reflexes, loss of long-latency reflexes, and changes in muscle properties [25]. According to the iBF theory, sensory input is sent back to the CNS to activate the impaired neuronal networks (biofeedback); in turn, the activated CNS enhances its descending signals [2]. Therefore, the spasticity could be ameliorated by HAL therapy. Furthermore, a previous study showed that HAL was effective for treating spastic hemiplegia due to stroke [16], and two studies have shown that HAL treatment for stroke patients facilitated cortical activities in the damaged brain [16, 17].

In this study, significant improvements were seen in gait ability following robotic rehabilitation. The results showed improvements in a series of clinical scales such as 10MWT, BI, GARS-M, and WISCI II. All participants showed improvements in gait ability that were similar to those of previous reports concerning the use of HAL for spine disorders such as SCI [8–14], SDAVF [41], and OPLL [18–22, 26]. It is noteworthy that participants in our study underwent surgery for various reasons such as spinal cord tumor, vascular disease, and bone degenerative disease. Additionally, clinical manifestations of vascular disease and tumors in the spine are similar [27, 28], and rehabilitation outcomes following vascular-related and traumatic SCI were reportedly not significantly different [29]. These facts may indicate that HAL therapy may be applied for a variety of disorders with spinal cord origins.

This study also showed the usefulness of HAL for postoperative rehabilitation. There have been only five case reports of HAL-assisted rehabilitation for a patient who underwent surgery for thoracic OPLL [18–22]. It is advantageous that HAL does not interfere with the skin incision and can be used for patients with a corset. In our experience, HAL was considered to facilitate early recovery after spine surgery.

In this study, HAL treatment was performed for patients with rare spinal diseases. In previous studies, the diagnosis and surgical management were emphasized rather than the rehabilitation programs, even though it has been considered that improvement after surgery depends on the length of time and initiation of neurological rehabilitation [30]. However, outcome measures have not been standardized. Previous reports showing the clinical outcomes of treatment for the same spine disorders are summarized in Table 2 [30–40].

We also reviewed clinical studies of gait training using HAL for spine disorders. A systematic search of the literature was conducted using the PubMed database. Search terms were “HAL” OR “Hybrid Assistive Limb” AND “Spinal Cord Injury” OR “OPLL.” We searched Google Scholar, and only one work [19] was included from that search. Studies only reporting HAL for gait training were included. Of 20 literatures, six were excluded due to the difference in the type of HAL robot. Overall, 14 studies met the inclusion criteria and were subject to critical review (Table 3) [8–14, 18–22, 26, 41].

Results of the systematic review revealed that HAL treatment was performed mainly for spinal cord injury and degenerative disease at various stages of disorders. Overall, these previous reports [12, 13, 19–22, 41] showed that both the speed and cadence were increased compared with our results. We considered the difference in the change in the gait speed. Previous reports [12, 13, 19–22, 41] indicated that more steps increased the cadence, whereas our results indicated that long steps decreased the cadence. Although natural recovery in the acute state following injury or surgical intervention has to be taken into account, it was thought that the functional recovery rate could be facilitated by HAL treatment from an early stage [18–20, 26].

Even though our study showed a significant improvement with HAL treatment, it had several limitations. We investigated a relatively small number of patients with heterogeneous characteristics. Our patients underwent HAL treatment during the early postoperative state, but our cohort did not have a control group. Therefore, it is possible that spontaneous recovery following surgery may have contributed to the postoperative course. However, it should also be noted that our patients experienced earlier recovery than those described in previous reports [18–20, 26] because our patients started HAL therapy during relatively early postoperative periods.

## 5. Conclusions

We showed the feasibility and safety of HAL treatment and determined that it could potentially facilitate functional recovery, even for postoperative patients. Further studies involving more patients and a control group are warranted to verify our findings.

## Figures and Tables

**Table 1:** Patient characteristics.

Patient	Age (years)	Sex	Diagnosis	Lesion level	Surgery-HAL interval (days)	Number of HAL sessions	Rehabilitation period (days)
1	48	Male	Arachnoid cyst	C5–Th1	7	3	7
2	65	Male	Dural AVF	Th6-7	13	12	35
3	56	Male	Dural AVF	Th6-7	10	3	4
4	70	Male	Cervical OPLL	C2–Th1	14	5	9
5	67	Male	Spinal lipoma	L2–5	21	5	7
6	72	Male	Cervical spondylosis	C4–5	11	5	10
7	29	Female	Spinal ependymoma	C6	29	4	18
8	36	Female	Spinal ependymoma	C2-3	14	3	21
9	39	Female	Spinal ependymoma	Medulla oblongata to Th1	19	5	11
Mean ± SD	53.6 ± 16.1				14.2 ± 8.1	5.0 ± 2.6	13.6 ± 9.1

AVF = arteriovenous fistula; OPLL = ossification of the posterior longitudinal ligament; SD = standard deviation.

**Table 2:** Review of reports of several postoperative spinal diseases.

Diagnosis	Author, year	Sample size, *n* (sex)	Age (years) (mean)	Follow-up (mean)	Outcome measures	Presenting symptoms	Rehabilitation program	Long-term results after surgical treatment
Arachnoid cyst	Bond et al., 2012 [31]	31 (M 14; F 17)	1–17 (8.1)	13 days to 12.6 years (4.4 years)	Categorized as complete remission, improvement, stable, or worse	Pain: 13 cases Lower extremity weakness: 12 cases Gait instability: 10 cases Spasticity: 6 cases Sensory loss: 3 cases Bladder dysfunction: 2 cases	N/A	21 patients had complete remission of symptoms 6 patients had symptom improvement 3 patients were stable 1 patient had worsened symptoms

Dural AVF	Behrens and Thron, 1999 [30]	21 (M 18; F 3)	33–75 (57.0)	5 months to 11 years (50 months)	Muscle strength	Flaccid: 9 patients Spastic: 12 patients	N/A	Improved: 67%; unchanged: 19%; deteriorated: 14%
					Walking distance	Wheelchair: 7 patients	N/A	Improved: 57%; unchanged: 29%; deteriorated: 14%
					Sensory loss	Sensory disturbance: 18 patients	N/A	Improved: 38%; unchanged: 62%
					Pain	No patient	N/A	Improved: 5%; unchanged: 71%; deteriorated: 24%
					BI (score)	70.5 ± 20.43 (range 20–95)	N/A	85.75 ± 13.81 (range 50–100)
	Van Dijk et al., 2002 [32]	49 (M 39; F 10)	28–78 (63)	12 days to 9.9 years (32.9 months)	Aminoff score of disability^∗^	The median gait score was 3 The median bladder score was 3	N/A	The median gait score was 2 The median gait score was 2

Cervical OPLL	Onari et al., 2001 [33]	30 (M 22; F 8)	34–61 (51.3)	10–23 years (14.7 years)	Okamoto's classification for the degree of walking disability^∗∗^	Grade a: no patient Grade b: 9 patients Grade c: 18 patients Grade d: 3 patients	N/A	Improvement: 2 grades in 16 patients Improvement: 1 grade in 8 patients Ambulatory deterioration in 6 patients
	Iwasaki et al., 2002 [34]	64 (M 43; F 21)	42–78 (56)	10–16 years (12.2 years)	JOA scoring system for cervical myelopathy^∗∗∗^	The mean preoperative total JOA score was 8.9 UE motor score: 2.5 ± 1.0 LE motor score: 2.2 ± 0.9 Sensory score: 2.2 ± 1.7 Bladder score: 2.2 ± 0.8	N/A	The mean last follow-up total JOA score was 13.8 UE motor score: 3.5 ± 0.8 LE motor score: 2.9 ± 1.1 Sensory score: 4.6 ± 1.4 Bladder score: 2.7 ± 0.5

Spinal lipoma	Lee et al., 1995 [35]	6 (M 3; F 3)	8–45 (27)	12–96 months (53.8 months)	Clinical and functional classification scheme^∗∗∗∗^	Grade I: no patient Grade II: 3 patients Grade III: 3 patients Grade IV: no patient	N/A	Grade І: 1 patient Grade II: 2 patients Grade III: 2 patients Grade IV: 1 patient

Cervical spondylosis	Wang et al., 2004 [36]	204 (M 145; F 59)	36–92 (63)	16 months	Nurick score for myelopathy^∗∗∗∗∗^	Score 0: 6 patients Score 1: 56 patients Score 2: 71 patients Score 3: 28 patients Score 4: 24 patients Score 5: 19 patients	N/A	Score 0: 64 patients Score 1: 72 patients Score 2: 31 patients Score 3: 10 patients Score 4: 19 patients Score 5: 8 patients
	Singh et al., 2009 [37]	50 (M 36; F 14)	56.7 ± 13.7	3 years	30-m walking test (sec)	53.6 ± 10.3	N/A	38.6 ± 6.9
	Kadaňka et al., 2011 [38]	64 (M, 46; F, 18)		12 years	10-m walking test (sec)		N/A	
		Without surgery: *n* = 32	Without surgery: 47–65 (54.5)	12 years	10-m walking test (sec)	7.0 (5.3; 10.7)^∗∗∗∗∗∗^	N/A	7.1 (5.1; 12.5)
		With surgery: *n* = 32	With surgery: 41–65 (51.0)	12 years	10-m walking test (sec)	8.0 (5.0; 29.8)	N/A	7.3 (5.1; 25.7)

Spinal ependymoma	Li et al., 2013 [39]	38 (M 19; F 19)	11–60 (35.3)	1 year	Modified McCormick classification^∗∗∗∗∗∗∗^	Grade I: 18 patients Grade II: 11 patients Grade III: 9 patients Grade IV: 0 patient	N/A	Grade I: 15 patients Grade II: 14 patients Grade III: 7 patients Grade IV: 2 patients
	Kaner et al., 2010 [40]	21 (M, 13; F, 8)	17–57 (34)	12–168 months (54 months)	Modified McCormick classification	Grade I: 11 patients Grade II: 5 patients Grade III: 3 patients Grade IV: 2 patients	They performed rehabilitation from an early postoperative day, but the content and method were not described at all	Grade I: 20 patients Grade II: 1 patient Grade III: 0 patient Grade IV: 0 patient


M = male; F = female; N/A = not available; AVF = arteriovenous fistula; BI = Barthel Index; OPLL = ossification of the posterior longitudinal ligament; JOA = Japanese Orthopaedic Association; UE = upper extremity; LE = lower extremity; the mean ± the standard deviation. ^∗^Aminoff score of disability (classification of gait disturbance: grade 1: leg weakness or abnormal gait and no restricted activity; grade 2: grade 1 with restricted activity; grade 3: requiring 1 stick or similar support for walking; grade 4: requiring 2 sticks or crutches for walking; and grade 5: unable to stand and confined to bed or wheelchair. Classification of micturition: grade 1: hesitance, urgency, or frequency; grade 2: occasional urinary incontinence or retention; and grade 3: total urinary incontinence or retention). ^∗∗^Okamoto's classification for the degree of walking disability (a: impossible to walk; b: walk with aids; c: independent walk with spasm; d: walk with difficulty, with or without spasm; e: walk easily but difficult to walk continuously; and f: normal or almost normal walking). ^∗∗∗^JOA scoring system for cervical myelopathy (motor function of fingers, shoulder and elbow, and lower extremity; sensory function of upper extremity, trunk, and lower extremity; and bladder function. Total score for a healthy patient = 17. Normal score of UE motor: 4, LE motor: 4, sensory: 6, and bladder: 3). ^∗∗∗∗^Clinical and functional classification scheme (grade I: neurologically normal, grade II: sensorimotor deficit affecting the function of the involved limb, grade III: more severe neurological deficit, and grade IV: severe deficit). ^∗∗∗∗∗^Nurick score for myelopathy (0: root involvement but no evidence of spinal cord disease; 1: spinal cord disease but no difficulty in walking; 2: slight difficulty in walking, but still employable; 3: difficulty in walking preventing full-time work or housework but independent ambulation; 4: able to walk with assistance or a walker; and 5: chair-bound or bedridden). ^∗∗∗∗∗∗^Median (5th–95th percentile range). ^∗∗∗∗∗∗∗^Modified McCormick classification (grade I: neurologically normal, normal ambulation and professional activity, and minimal dysesthesia; grade II: mild motor and sensory deficit, independent function, and ambulation maintained; grade III: moderate sensorimotor deficit, restriction of function, and independent with an external aid; grade IV: severe sensorimotor deficit, restricted function, and dependent; and grade V: paraplegia and quadriplegia and even/flickering movement).

**Table 3:** Review of HAL treatment for spinal disease.

Author, year	Age (years), sex	Diagnosis	Time when starting HAL	Number of HAL sessions	10MWT Speed (m/min)		10MWT Cadence (steps/min)		BI		WISCI II	
					Pre	Post	Pre	Post	Pre	Post	Pre	Post
Our result	53.6 ± 16.1, M 6; F 3	Table 1	POD 14.2 (7–29 days)	2-3 times/week, 5.0 ± 2.6	64.1 ± 16.0	74.8 ± 10.8	102.7 ± 17.6	92.7 ± 10.9	83.3 ± 16.0	95.6 ± 5.8	19.7 ± 0.5	20.0 ± 0.0

Sakakima et al., 2013 [18]	60, F	T2–8 OPLL, T9–10 OLF	POD 49 (after bed rest) to 15 weeks	6 times/week, 48	N/A	N/A	N/A	N/A	N/A	N/A	0	8

Kubota et al., 2016 [19]	43, M	T8–11, L1–3 OPLL	POD 14–44	2-3 times/week, 10	Approximately 20^∗^	Approximately 50^∗^	Approximately 42^∗^	Approximately 85^∗^	60	85	13	16

Fujii et al., 2017 [20]	63, F	T3–7 OPLL	POD 44 (after bed rest) to POD 73	2-3 times/week, 10	15.9	31.8	43.8	77.9	N/A	N/A	8	16

Kubota et al., 2017 [21]	66, F	C5-6 OPLL	14 years after surgery	Once/2 weeks, 10	22.5	46.7	61.9	81.6	N/A	N/A	16	16

Shimizu et al., 2017 [41]	48, M	T12–L1 SDAVF	6 months after surgery	2 times/week, 10	Approximately 13^∗^	Approximately 29^∗^	Approximately 39^∗^	Approximately 62^∗^	N/A	N/A	7	12

Taketomi et al., 2018 [22]	70, M	T9–12, L2/3, L5 OLF, C3–7 OPLL	1 year after his third surgery	2 times/week, 10	49.8	58.2	109.8	120	N/A	N/A	N/A	N/A

Puentes et al., 2018 [26]	59.6 ± 13.9, M 2; F 3 (acute group)	OPLL	POD 24.4 (15–32 days)	2 times/week, 10	Approximately 28^∗^	Approximately 54^∗^	N/A	N/A	Approximately 63^∗^	Approximately 83^∗^	N/A	N/A
	70.1 ± 6.9, M 7 (chronic group)		POD 1151.4 (287–3655 days)		Approximately 48^∗^	Approximately 56^∗^	N/A	N/A	Approximately 100^∗^	Approximately 100^∗^	N/A	N/A

Aach et al., 2014 [9]	48 ± 9.4, M 6; F 2	T8–L2 SCI	8.1 (1–19) years posttrauma—90 days	5 times/week, 51.75 ± 5.6	0.28 ± 0.28 (m/sec)	0.5 ± 0.34	N/A	N/A	N/A	N/A	10 ± 4.3	11.1 ± 3.7

Cruciger et al., 2016 [10]	52, M	L3 SCI	10 years posttrauma—12 weeks	5 times/week, mean 54.5	85.6 ± 56.9 (sec)	44.3 ± 34.6	N/A	N/A	N/A	N/A	N/A	N/A
	40, F	L1 SCI	19 years posttrauma—12 weeks									

Sczesny-Kaiser et al, 2015 [11]	46.9 ± 2.7, M 7; F 4	T8–L2 SCI	8.8 (0.7–17) years postinjury—12 weeks	5 times/week, 60	0.25 ± 0.05 (m/sec)	0.5 ± 0.07	N/A	N/A	N/A	N/A	N/A	N/A

Watanabe et al., 2017 [12]	62, M	T12–L1 SCI	7 days post onset	3-4 times/week, 7-8	21.6	39.6	62	99.5	35	60	5	18
	61, F	T8–10 SCI	14 days post onset	3-4 times/week, 7-8	21.6	60.7	52	97.3	60	80	4	13

Grasmücke et al., 2017 [8]	44.3 ± 13.9, M 43; F 12	C2–L4 SCI	6.9 (1–22) years posttrauma—12 weeks	5 times/week, 60	70.45 ± 61.5 (sec)	35.22 ± 30.8	N/A	N/A	N/A	N/A	9.35 ± 5.12	11.04 ± 4.52

Jansen et al., 2017 [13]	48 ± 9.4, M 6; F 2	T8–L2 SCI	8.1 (1–19) years posttrauma—12 weeks	5 times/week, 51.75 ± 5.6	0.28 ± 0.10 (m/sec)	0.5 ± 0.12	41.85 ± 9.45	56.7 ± 9.9	N/A	N/A	10 ± 1.5	11.13 ± 1.3
	Subgroup 1: *n* = 4	T8–L2 SCI	Plus 40 weeks (at 52 weeks)	3–5 times/week, 126.8 ± 7.9	28.61 ± 6.9 (sec)	21.22 ± 6.6	49.71 ± 8.8	72.16 ± 6.9	N/A	N/A	No patient improved or worsened	
	Subgroup 2: *n* = 4	T8–L2 SCI	Plus 40 weeks (at 52 weeks)	Once/week, 32.3 ± 3.3	34.28 ± 18.2 (sec)	34.61 ± 17.3	63.65 ± 18.7	62 ± 18.8	N/A	N/A	No patient improved or worsened	

Jansen et al., 2018 [14]	44.8 ± 13.8, M 15; F 6	C4–L3 SCI	6.5 (1–19) years posttrauma—12 weeks	5 times/week, 60	61.17 ± 44.27 (sec)	32.18 ± 25.53	30.9 ± 8.71 (number of steps)	20.7 ± 5.51	N/A	N/A	10.7 ± 4.95	11.7 ± 4.5

M = male; F = female; 10MWT = 10-m walking test; BI = Barthel Index; WISCI II = the walking index for spinal cord injury; POD = postoperative day; Pre = before training; Post = after training; N/A = not available; OPLL = ossification of the posterior longitudinal ligament; OLF = ossification of ligamentum flavum; SDAVF = spinal dural arteriovenous fistula; SCI = spinal cord injury. ^∗^Estimated from the presented figure in the paper.

## Data Availability

The data used to support the findings of this study are available from the corresponding author upon request.
